# The Photoisomerization Pathway(s) of Push–Pull Phenylazoheteroarenes**

**DOI:** 10.1002/chem.202002321

**Published:** 2020-10-14

**Authors:** Sergi Vela, Clémence Corminboeuf

**Affiliations:** ^1^ Institute of Chemical Sciences and Engineering Laboratory for Computational Molecular Design École Polytechnique Fédérale de Lausanne (EPFL) 1015 Lausanne Switzerland

**Keywords:** azo-based photoswitches, azoheteroarene derivatives, conical intersection energies, push–pull character, *trans*–*cis* isomerization

## Abstract

Azoheteroarenes are the most recent derivatives targeted to further improve the properties of azo‐based photoswitches. Their light‐induced mechanism for *trans*–*cis* isomerization is assumed to be very similar to that of the parent azobenzene. As such, they inherited the controversy about the dominant isomerization pathway (rotation vs. inversion) depending on the excited state (nπ* vs. ππ*). Although the controversy seems settled in azobenzene, the extent to which the same conclusions apply to the more structurally diverse family of azoheteroarenes is unclear. Here, by means of non‐adiabatic molecular dynamics, the photoisomerization mechanism of three prototypical phenyl‐azoheteroarenes with increasing push–pull character is unraveled. The evolution of the rotational and inversion conical intersection energies, the preferred pathway, and the associated kinetics upon both nπ* and ππ* excitations can be linked directly with the push–pull substitution effects. Overall, the working conditions of this family of azo‐dyes is clarified and a possibility to exploit push–pull substituents to tune their photoisomerization mechanism is identified, with potential impact on their quantum yield.

## Graphical abstract

**At a push**: Azoheteroarene derivatives with increased push–pull character have been investigated computationally, revealing how the push–pull substitution affects the rotation and inversion conical intersection energies, the preferred pathway, and the associated kinetics upon both nπ* and ππ* excitations.

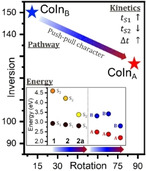

## Introduction

Molecular photoswitches can alter their chemical/biological functions by undergoing conformational, configurational, or structural changes upon application of light. Although nature exploits them to trigger key processes in living organisms, the synthetic analogs are increasingly used as memory devices,[^1^, ^2^, ^3^] actuators,^[4]^ sensitizers,^[5]^ and sensors.^[6]^ Dyes based on the azo group are among the most investigated, the archetype being azobenzene (AB).[^7^, ^8^] Its *cis*–*trans* photoisomerization is highly appreciated owing to its significant structural change.^[4]^ Also, AB has multiple functionalization sites, which led to the development of derivatives exhibiting improved thermal stability and visible light absorption.^[9]^ All these advances fostered its application in the nascent field of photo‐pharmacology.^[10]^ Azoheteroarenes are the most recent derivatives investigated in the quest of better azo‐based photoswitches.^[11]^ Their main advantage with respect to AB relies upon their greater electronic and structural diversity, which allow for interesting functionality in their backbone. Examples are the possibility to achieve T‐shaped *Z*‐isomer structures with longer half‐life times and better photostationary distributions,^[12]^ the modulation of the hydrazone tautomerism to further tune their kinetics,^[13]^ or the addition of metal‐coordinating sites, which makes them ideal candidates to trigger spin transitions.[^14^, ^15^]

In AB, the photoswitch is triggered upon excitation to either of the productive nπ* and ππ* states (typically, S_1_ and S_2_). For decades, there has been controversy about its isomerization mechanism[^7^, ^16^] with conflicting experimental[^17^, ^18^, ^19^, ^20^, ^21^] and theoretical[^22^, ^23^, ^24^, ^25^, ^26^] reports. The current consensus is that the photoisomerization occurs once the molecule is in S_1_, through an S_1_/S_0_ conical intersection (CoIn) with either rotational or inversion character. Specifically, it has been proposed that these CoIn are the extremes of a crossing seam connecting S_1_/S_0_, with rotation‐ (inversion‐) like structures at its lower (higher) energy end.[^24^, ^27^] The preferred pathway depends on the excitation energy,^[28]^ solvent,^[28]^ pressure,^[29]^ temperature,[^30^, ^31^] and has an impact on the quantum yield: AB photoisomerizes with a higher quantum yield when excited to the nπ* than to the ππ* state,^[30]^ which is attributed to the increased accessibility of the rotational pathway.[^7^, ^32^, ^33^]

Azoheteroarenes are expected to follow a similar mechanism, but the picture is much less complete.^[11]^ So far, their photoisomerization has been investigated in systems based on indole,^[3]^ pyridine,[^34^, ^35^] pyrimidine,^[36]^ and thiazole.^[37]^ These few cases suggest that the structural diversity of azoheteroarenes contributes to create a similar level of complexity as in the AB derivatives. A major limitation of these investigations is that the experiments do not provide direct information on the relaxation pathways, whereas computations are often limited to exploring predefined regions of the ground‐ and low‐lying excited states potential energy surfaces (PES).[^3^, ^34^, ^35^, ^36^, ^37^] Although this picture is informative, it is still insufficient to ascertain the effect of temperature and excitation energy on the chosen pathway, and hence on the photoisomerization quantum yield and kinetics. These are important aspects that deserve deeper computational analyses, which more closely mimic the actual experimental conditions.

## Results and Discussion

**Figure 1:**
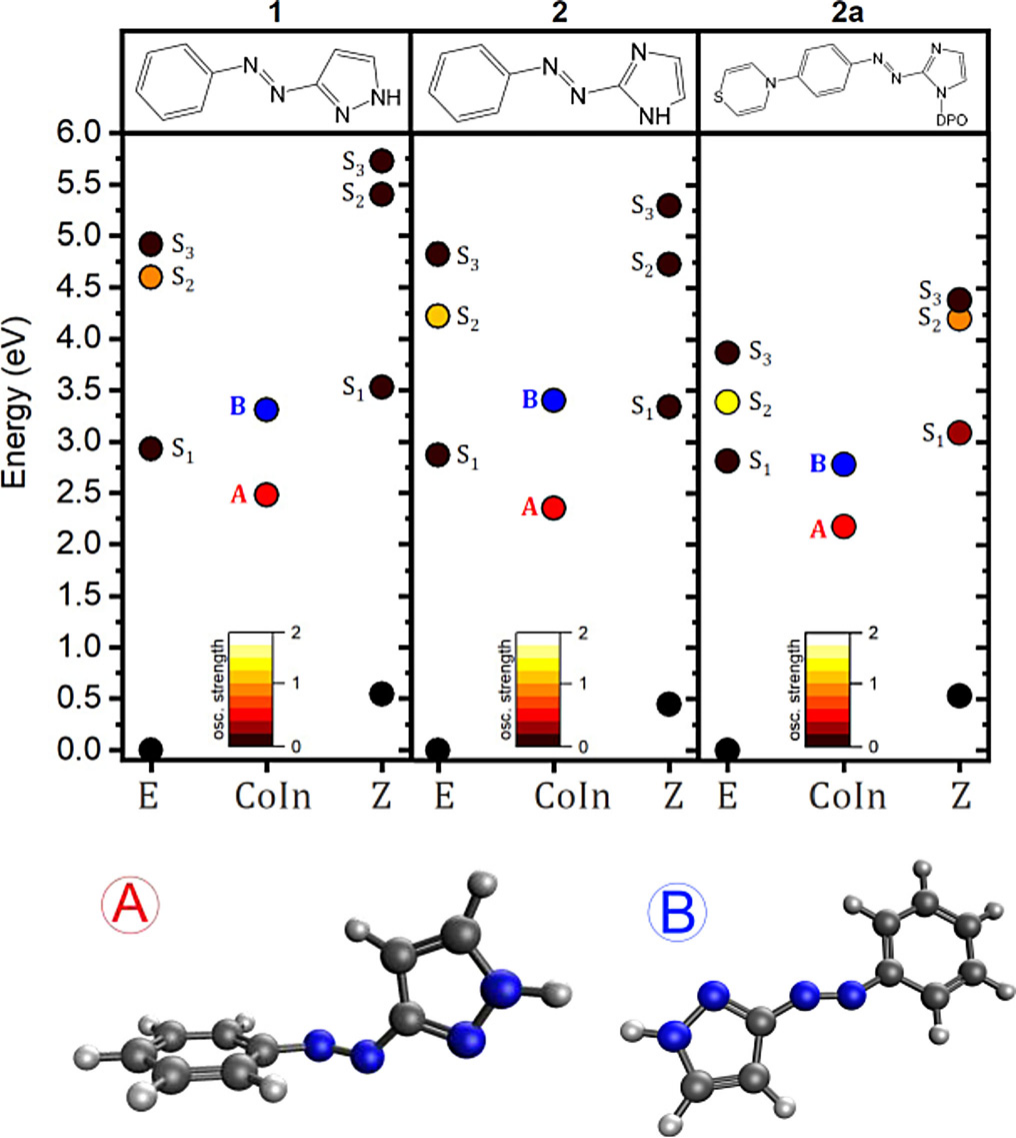
(Top) Relative energy of CoIn_A_ and CoIn_B_ with respect to the *E*‐ and *Z*‐minima, and their S_1–3_ excitations at FC, the oscillator strength of which is displayed in the color code. All computations have been carried out at the ωB97X‐D/6‐31G(d) level. Raw data is in Tables S1.1 and S2.1 in the Supporting Information. (Bottom) Structure of CoIn_A_ and CoIn_B_ for compound **1**.

In this computational work, we analyze the *E*‐to‐*Z* photoisomerization of three phenylazoheteroarenes: the unsubstituted 3‐pyrazole (**1**) and 2‐imidazole (**2**), and a derivative of the latter (**2 a**), featuring DPO (2,5‐diphenyl‐1,3,4‐oxadiazole) and thiazine as the phenyl and heteroarene substituents, respectively (see Figure 1). Heteroarenes have an increased push–pull character, by virtue of the stabilization of a resonant form.^[11]^ Such stabilization is progressively stronger in compounds **1**, **2**, and **2 a**, which has important consequences on their thermal stability, and on the energy and nature of their productive nπ* and ππ* transitions.^[38]^ Specifically, their properties are systematically found at the edge of the explored values derived from the screening of 512 phenylazoheteroarenes. This can be taken as an indication that these compounds are representative of the structural and electronic diversity present within phenylazoheteroarenes. What remains to be known is the impact of the increase in push–pull character on the photoisomerization mechanism. A literature survey of push–pull AB derivatives reveals conflicting reports, with computational PES analyses favoring rotation as the single relaxation channel^[39]^ (B3LYP/6‐31G* level), or rotation and inversion upon S_1_ and S_2_ excitation, respectively^[40]^ (CAS(6,5)/4–31G level), and experiments (fluorescence^[41]^ and absorption spectroscopy[^42^, ^43^]) favoring a unimodal relaxation through rotation. Yet, reported works on the topic remain scarce, and extrapolation to the realm of azoheteroarenes uncertain. Such a generalization is especially relevant given the possibility to use push–pull substituents to tune the photoisomerization quantum yield of azo‐dyes. With this in mind, our goal is to identify the isomerization pathways, and the associated kinetics, of **1**, **2**, and **2 a** upon excitation to the *productive* nπ* and ππ* states.

**Figure 2:**
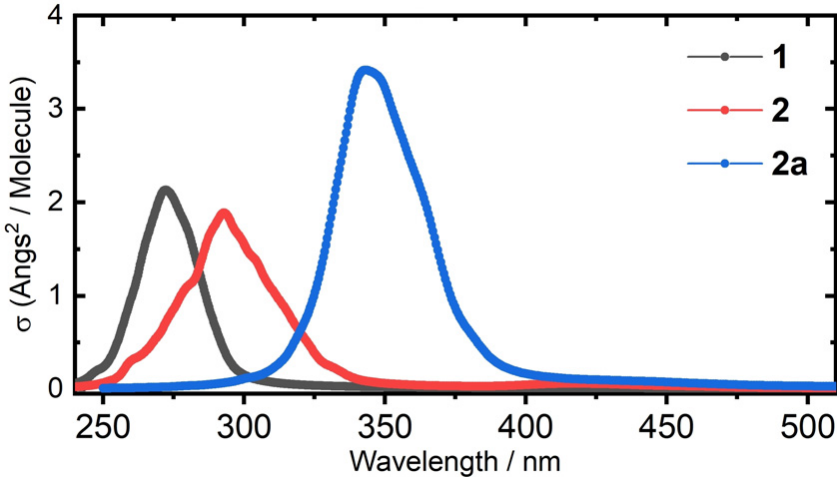
Computed absorption spectrum of **1**, **2**, and **2 a** at the ωB97X‐D/6‐31G(d) level.

The initial assessment of the vertical nπ* (S_1_) and ππ* excitations (S_2_) of **1**–**2 a** at the respective *E*‐isomer minima (Figure 1) shows no significant difference between **1** and **2**, whereas the ππ* is significantly redshifted in **2 a** by the increased charge‐transfer character from the thiazine to the azo group.^[38]^ The shift is also clearly visible in the absorption spectra computed at the same level (i.e., ωB97X‐D/6‐31G(d) level, see Figure 2 and Computational Details), which includes the conformational and vibrational transitions with the Nuclear Ensemble (NE) approach.^[44]^

The ground, nπ*, and ππ* states are connected with each other through CoIn. At the CASSCF (i.e., SA3‐CASSCF/6‐31G*) level, three CoIn were identified for (unsubstituted) phenylazoindole photoswitches.^[13]^ These are: (*i*) CoIn_A_, characterized by a CNNC torsion angle close to 90° (corresponding to the rotation), (*ii*) CoIn_B_, which involves quasi‐linear NNC angles (characteristic of an inversion), and (*iii*) CoIn_C_, which features an intermediate torsion, longer N=N distance, and CNN angles close to 100°. The former two (CoIn_A_ and CoIn_B_) connect the PES of the ground (GS) and nπ* states, whereas the latter connects the nπ* and ππ* surfaces. CoIn_A_ was found below the nπ* excitation energy at Franck–Condon (FC), whereas CoIn_B_ is higher, and hence only accessible after excitation to ππ* or above. Accordingly, it was proposed that excitation to nπ* leads to CoIn_A_, whereas excitation to ππ* leads to CoIn_C_ (ππ*/nπ*) and to CoIn_B_ (nπ*/GS) before reaching the GS.^[13]^ The former pathway would lead to a higher quantum yield (QY) than the latter.^[13]^ Such characterization is very similar to what is known for AB,[^24^, ^27^, ^45^] except for the proposed non‐planar (i.e., twisted geometry) CoIn_C_: the ultrafast decay from ππ* to nπ* in AB,[^19^, ^20^, ^46^] suggests a structure close to the planar FC geometry instead.

The CoIn_A_ and CoIn_B_ of **1**, **2**, and **2 a** were characterized here with the Tamm–Dancoff approximation (TDA) and ωB97X‐D (see the Supporting Information for complementary ADC(2) computations) by using a static CoIn search method (see Computational Details).^[47]^ Their structures feature the characteristic CNNC torsion (close to 90°) and NNC (quasi‐ linear) bending, respectively (see Table S2.1 in the Supporting Information). The energy of CoIn_A_ decreases by about 0.3 eV with the increase in push–pull character (see Figure 1). We associate this shift to the reduction of the azo N=N bond‐order (see the Wiberg index (WI) in Table S2.1 in the Supporting Information) as a longer N=N distance facilitates the CNNC rotation towards CoIn_A_. As expected (see above), CoIn_A_ is found below the S_1_ excitation energy at FC. In turn, CoIn_B_ lies at about 3.3 eV from the respective *E*‐minima, and slightly above the S_1_ excitation energy at FC. An exception is **2 a**, for which CoIn_B_ and S_1_ are almost degenerate. That suggests the opening of the inversion pathway upon S_1_ excitation, in contradiction to the expected mechanism. Overall, the two sets of CoIn reported herein (A and B) present very similar structural features to those reported in the existing literature for AB^[22]^ and azoheteroarene derivatives.[^13^, ^35^] None of the levels tested herein (i.e., ωB97X‐D, ADC(2)) were able to locate the CoIn_C_ as proposed in Ref. [13], not even for the same phenylazoindole. Although the identification of CoIn_C_ may be an artefact from SA3‐CASSCF/6‐31G* (see below),^[48]^ TDA‐ωB97X‐D describes correctly the PES regions that correspond to the two dominant photoisomerization pathways. As such, we are confident that more sophisticated simulations based on molecular dynamics can be pursued at this level (see Computational Details).

A swarm of Non‐Adiabatic Molecular‐Dynamics (NAMD) trajectories based on Tully surface‐hopping were initiated at the nπ* and ππ* states for **1**, **2**, and **2 a**, and propagated for a maximum of 1000 fs, or until an S_1_–S_0_ energy gap below 0.1 eV is reached (see Computational Details). In the latter case, it is assumed that population transfer to the ground state will occur, leading to either of the two minima (*E* or *Z*). Note that although the termination criterion does not presuppose the character of S_1_ (nπ* or ππ*), in practice, however, S_1_ is the nπ* state for all terminated trajectories. In general, we favor the nomenclature nπ*/ππ* to specifically refer to these states, and use the S_1_–S_2_ nomenclature when the state character is not relevant, only the order.

**Figure 3:**
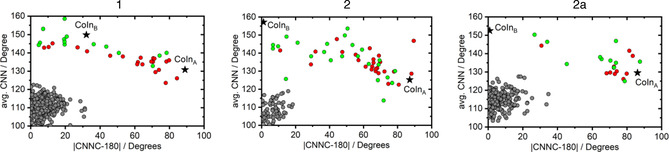
Space of CNN and CNNC angles featured by the relevant geometries in all trajectories of **1**, **2**, and **2 a**. The CNNC angle is evaluated as the deviation from planarity, with 0° corresponding to the *E*‐isomer, and 90° corresponding to CNNC either +90 or −90 degrees. In color, the geometries that reached an S_1_/S_0_ CoIn before the time limit (1 ps). The color code indicates trajectories initiated at S_1_ (red) and S_2_ (green). The black stars indicate the geometry of the CoIn obtained from static computations. In dark gray, all geometries at which a hopping between S_1_ and S_2_ occurred in the NAMD trajectories. All computations have been performed at the ωB97X‐D/6‐31G(d) level, and the quasi‐degeneracy along the crossing seam is verified by additional CC2 and ADC(2) computations with the TZVP basis set (see Section S3 in the Supporting Information).

Figure 3 shows the structure at which state crossings occur based on the two main variables: the CNN angle and the CNNC torsion measured as the deviation from planarity (i.e., |CNNC−180|. We chose this CNNC metric as a result of **1**–**2 a** having no particular preference towards a clockwise or counterclockwise rotation about the CNNC dihedral, which results in a similar distribution of positive and negative CNNC dihedral angles. This is in contrast to some reported heteroarenes featuring a stereospecific relaxation mechanism.[^49^, ^50^] In **1**–**2 a**, the relaxation from the ππ* to the nπ* state occurs at flat geometries similar to the *E*‐isomer minimum (see gray circles). As mentioned before, flat geometries have been invoked to explain the ultrafast ππ*→nπ* decay in AB.[^19^, ^46^] This point is thus reinforced by our simulations, and its validity seems to be extended to azoheteroarenes albeit in contradiction with the proposed non‐planar CoIn_C_ of phenylazoindoles (see also Section S2 in the Supporting Information).^[13]^ The CoIn connecting the nπ* and ground states combine both CNN inversion and CNNC torsion (see colored circles). In fact, the distribution of CoIn describes a crossing seam (as in AB[^24^, ^27^]), with CoIn_A_‐ and CoIn_B‐_like structures at the extremes (see Figure 3, Figure S3.4, and Table S3.2 in the Supporting Information). CoIn_B_ being higher in energy (see Figure 1), the inversion pathway is more often (but not exclusively) followed upon excitation at S_2_, whereas excitation to S_1_ predominantly leads to a rotational mechanism (see Figure 1). There are, however, many trajectories in which CoIn_B_‐like (CoIn_A_‐like) are reached upon excitation to S_1_ (S_2_), which indicates that the excitation energy does not completely discriminate between photodeactivation pathways. This point might, in fact, be at the heart of the controversy about the dominant mechanism in AB and azoheteroarene derivatives, and their strong dependency on external factors such as temperature, pressure, or solvent. Finally, the vast majority of CoIn in **2 a** are rotational (see Figure 3). The reason is that the nπ* and GS PES are no longer quasi‐degenerate in a region of the crossing seam associated with the inversion pathway (see Figure S3.4 in the Supporting Information). This explains why push–pull derivatives favor the rotational over the inversion pathway. This outcome could have not been anticipated from the energy maps in Figure 1. The energy of S_1_ and CoIn_A_ for **2 a** are similar to those of **1** and **2**, and the lower S_2_ excitation energy is counterbalanced by a more stable and, thus, equally accessible CoIn_B_. The low ratio of inversion‐like CoIn in **2 a** is thus not suggested by the static picture.

**Scheme 1:**
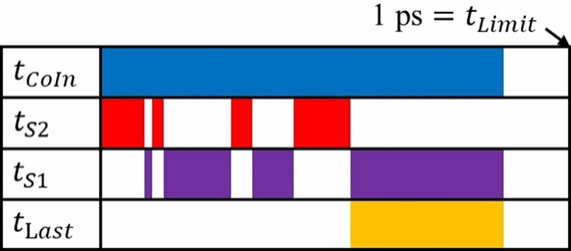
Schematic representation of the four characteristic times discussed in the main text to analyze the NAMD. These are computed as an average for each set of trajectories representing the S_1_ or S_2_ excitation of **1**–**2 a**.

In addition to the structural aspects at the crossing points, we analyze the time at which they are reached in the NAMD simulations. Overall, the CoIn connecting the nπ* and ground states in **1**, **2**, and **2 a** are reached in approximately 500 fs, with significant differences depending on the compound and excitation energy (see $t_{CoIn}$ in Table 1). The main steps are (*i*) the relaxation from the ππ* to the nπ* state, and (*ii*) the change in CNN and CNNC angles necessary to reach the crossing seam (see discussion above). The mechanism and kinetics of each individual steps is better understood by considering four characteristic times (see Scheme 1): $t_{CoIn}$ is the time until reaching a CoIn, *t* ${}_{S{}_{1}}$ and *t* ${}_{S{}_{2}}$ are the times spent in the S_1_ and S_2_ states, and $t_{Last}$ is the time required to reach a CoIn after the last crossing to S_1_. The difference between *t* ${}_{S{}_{1}}$ and $t_{Last}$ (Δt in Table 1) reveals whether the system is retained in a region of the S_1_‐PES with frequent crossings between S_1_ and S_2_ states, before it reaches a CoIn (see Computational Details).

**Table 1:** (Top) Ratio (*R*) of trajectories reaching a CoIn before the time limit (1 ps). (Bottom) The characteristic times described in the main text and in Scheme 1 (in fs). Confidence intervals associated with these values, as well as their convergence with the number of trajectories is given in Section S3.3 (in the Supporting Information).

	Initial State	Compound		
		**1**	**2**	**2 a**
*R*	S_1_	0.84	1.00	0.55
	S_2_	0.56	0.96	0.55
$t_{CoIn}$	S_1_	419	330	644
	S_2_	530	373	483
*t* ${}_{S{}_{1}}$	S_1_	419	330	637
	S_2_	250	260	413
*t* ${}_{S{}_{2}}$	S_1_	0	0	23
	S_2_	280	113	69
$t_{Last}$	S_1_	419	330	536
	S_2_	244	258	323
Δt	S_1_	0	0	100
	S_2_	6	2	90

Both $t_{CoIn}$ and *t* ${}_{S{}_{2}}$ depend on the relative energy separation between the nπ* and ππ* states; in general, *t* ${}_{S{}_{2}}$ decreases together with the energy gap. The stronger the push–pull character of a system, the more redshifted is the ππ* state, resulting in a smaller gap, and a faster decay from the ππ* to the nπ* state (see *t* ${}_{S{}_{2}}$ in Table 1). It is, however, interesting to observe that the overall photoisomerization process ($t_{CoIn}$ ) does not follow the same trend as *t* ${}_{S{}_{2}}$ , and this is for two reasons. In **1** and **2**, the energy gap between the nπ* and ππ* states is sufficiently large so that the relaxation from the ππ* state is a one‐way process. In other words, the trajectories proceed undisturbed along the nπ* PES towards a CoIn. In **2 a**, however, the two PES overlap more often, which leads to an increased probability of hopping back to the ππ* state, effectively delaying the evolution towards a CoIn. This is quantified by Δt in Table 1, and can also be verified in the time‐evolution of the S_2_ population (Figure S3.1 in the Supporting Information).

The second reason for the slower photoisomerization of **2 a** is that its trajectories need longer times to reach a CoIn once in the nπ* surface, as quantified by $t_{Last}$ (Table 1).^[51]^ Such a behavior is surprising if one considers that the energetic profile summarized in Figure 1 places the S_1_ excitation significantly higher in energy than CoIn_A_. Along this line, the PES of the nπ* state shows no energy barrier between the FC region and CoIn_A_ (see Figure S2.1 in the Supporting Information). The actual explanation for the longer $t_{Last}$ in **2 a** is rooted in the CoIn structure distribution within the crossing seam. Generally, the trajectories that reach a CoIn with a pronounced rotational character display slower kinetics than those with a marked tendency toward inversion (see Figure S3.2 in the Supporting Information). This difference is a manifestation of the distinct timescale associated with the rotation and inversion towards the crossing seam. In particular, the inversion‐like region of the seam is explored readily after populating S_1_. If a CoIn is not accessed therein, as in **2 a**, the system then evolves toward the rotation‐like region, exhibiting slower photoisomerization kinetics. This perspective is in agreement with what has been characterized computationally for phenylazoheteroarenes,^[37]^ namely that the evolution along the nπ* surface implies an initial flattening of the CNN angle (i.e., inversion), followed by the CNNC torsion (i.e., rotation). To summarize, push–pull derivatives undergo a faster decay from S_2_ to S_1_ but a slower evolution from S_1_ to S_0_ because of both the hopping back to ππ* and the longer time needed to reach the rotational CoIn. The clear preference of **2 a** for the rotational pathway potentially suggests that the increase in push–pull character may result in a photoisomerization process with a higher quantum yield (see above).

## Conclusion

We have characterized the static and dynamic photoisomerization pathway of three heteroarene derivatives (**1**–**2 a**). The static CoIn‐search reports rotation‐like (CoIn_A_) and inversion‐like (CoIn_B_) conical intersections connecting the nπ* and ground states at around 2.3 and 3.3 eV above the *E*‐minima, respectively. The results from the NAMD describe a crossing seam connecting CoIn_A_ and CoIn_B_, similar to what has been reported for AB.[^24^, ^27^] The decay from the ππ* to the nπ* state is more controversial. The non‐planar CoIn_C_ located^[13]^ by CASSCF for phenylazoindoles could not be identified in **1**–**2 a** (see Section S2 in the Supporting Information). However, the ultrafast (ca. 100 fs) relaxation observed from the ππ* to the nπ* state that proceeds at planar geometries close to the *E*‐isomer minimum, is in agreement with the literature on AB (100–300 fs).[^19^, ^52^, ^53^] The existence of CoIn_C_ may thus be an artefact from SA3‐CASSCF/6‐31G*.

With the increase in push–pull character, the ππ* state of the heteroarene is progressively redshifted, leading to a stronger overlap with the nπ* state, which speeds up the decay towards nπ*. Once in the nπ* state, further 200–600 fs are necessary to reach the crossing seam connecting the nπ* and ground states, close to the values reported for AB (ca. 500–1000 fs).[^7^, ^18^, ^19^, ^21^] The actual amount of time depends on which region of the crossing seam is accessed, with the rotational mechanism displaying a slower nπ*‐to‐GS relaxation. The unsubstituted heteroarenes (**1** and **2**) exploit both pathways, with rotation and inversion being slightly preferred upon excitation to the nπ* and ππ* states, respectively. In contrast, the push–pull derivative **2 a** exhibits a clear preference towards the rotational pathway upon excitation to both states, resulting in a slower photoisomerization than **1** and **2** as the process in **2 a** is further slowed down by population transfer back to the ππ*. Overall, push–pull derivatives feature a faster decay from ππ* to nπ*, but a slower one from nπ* to the ground state.

From a design perspective, push–pull derivatives may thus represent an appealing alternative to improve the photoisomerization quantum yields by virtue of its marked preference for the rotational pathway. It is worthwhile noting that that such preference could not be anticipated based on the energy maps (Figure 1). This mismatch, as well as the significant differences between the static and dynamic pictures at describing the crossing region (CoIn vs. crossing seam), highlights the risk of establishing conclusions on the photoisomerization mechanism based on the energy of the relevant points on the PES, as commonly done.

## Computational Details

Minimal energy crossing points were computed with CIOPT^[47]^ interfaced with Gaussian 09 (G09).^[54]^ Based on previous benchmarks,[^38^, ^55^, ^56^] we used TD‐DFT within the Tamm–Dancoff approximation (TDA), the ωB97X‐D functional,[^57^, ^58^] and the 6‐31G(d) basis set. The Non‐Adiabatic Molecular Dynamics (NAMD) simulations were performed with Newton‐X[^59^, ^60^] interfaced with G09.^[54]^ Additional computations at the ADC(2)/TZVP level can be found in the Supporting Information. The initial conditions were generated from the Wigner distribution based on the harmonic oscillator, five states (S_0_–S_4_), a Lorentzian broadening of 0.1 eV, an anharmonicity factor of 3, and at *T*=300 K. From these initial conditions, we obtain the (*i*) absorption spectra and (*ii*) a set of the geometries and velocities that could initiate the trajectories. The selected initial conditions are those in which the S_1_ and S_2_ excitation energy is centered (and within +/−0.1 eV) at the peak of the respective transition in the spectrum (see Figure 2). A swarm of 25 trajectories has been initiated at each S_1_ and S_2_ for **1** and **2** (see Section 2.1 in the Supporting Information). Owing to the much larger size of **2 a**, we reduced the number of trajectories to 20. Hence, a total of 140 trajectories were run.

The trajectories were computed by using TD‐DFT (within TDA) at the ωB97X‐D/6‐31G(d) level. NAMD were simulated with the fewest‐switches surface hopping^[61]^ corrected for decoherence effects (*α*=0.1 Hartree).^[62]^ Time‐derivative couplings^[63]^ were computed for all states except S_0_, which is excluded due to the difficulties of TDA to describe the multi‐reference character of the electronic wavefunction near a S_1_–S_0_ CoIn. Moreover, such limitations imply that the trajectories must be terminated right before the conical intersection is reached, which implies that photoisomerization quantum yields cannot be quantified. Accordingly, trajectories ran for a maximum of 1000 fs or until an S_1_–S_0_ energy gap below 0.1 eV is reached. In the latter case, it is assumed that the actual CoIn is very similar to the final geometry explored in the trajectory, and that it should be reached immediately after in time. The selected time limit of 1000 fs is sufficient to allow most of the trajectories to reach the CoIn (see Table 1). Trajectories are propagated in the microcanonical NVE ensemble. Evolution of the kinetic and potential energy for each set of trajectories is shown in Figure S3.6 (in the Supporting Information). Integration was done with a time step of 0.5 (0.025) fs for the classical (quantum) equations. This setup has been successfully employed to study other small‐size organic molecules.[^64^, ^65^, ^66^, ^67^, ^68^]

Surface‐Hopping Molecular Dynamics exploit statistics to mimic the dynamics of nuclear wavepackets,[^69^, ^70^, ^71^, ^72^] and hence we analyze them as a whole. The kinetics are assessed by using the characteristic times defined in Scheme 1. These are computed for each S_1_ and S_2_ excitation of **1**–**2 a** as an average using the trajectories that reach a CoIn before the time limit of 1000 fs. Should the trajectories be allowed to continue beyond 1000 fs, the associated times would change, $t_{CoIn}$ and *t* ${}_{S{}_{1}}$ would increase as the slower trajectories would start counting towards the average, whereas the change in *t* ${}_{S{}_{2}}$ is harder to anticipate. As a general rule, the values are more representative when the ratio of trajectories that reached a CoIn within the time limit is closer to 1 (*R* in Table 1).

Dataset: The dataset will be available upon publication at the Zenodo repository.

## Conflict of interest

The authors declare no conflict of interest.

## Supporting information

As a service to our authors and readers, this journal provides supporting information supplied by the authors. Such materials are peer reviewed and may be re‐organized for online delivery, but are not copy‐edited or typeset. Technical support issues arising from supporting information (other than missing files) should be addressed to the authors.

Supplementary
