# Supplementary material for: The Photoisomerization Pathway(s) of Push–Pull Phenylazoheteroarenes
Source: Chemistry. 2020 Oct 14;26(64):14724–9. doi: 10.1002/chem.202002321 (PMC7756763; doi:10.1002/chem.202002321)
Supplement: Supplementary file 1 — Supplementary [file CHEM-26-14724-s001.pdf]

Chemistry–A European Journal

Supporting Information

The Photoisomerization Pathway(s) of Push–Pull Phenylazoheteroarenes**

Sergi Vela and Clémence Corminboeuf*[a]

S1. Absorption Spectra and State Character

Absorption Spectra

Table S1.1 Energy, oscillator strength, and nature of the $S_{1\text{-}4}$ excitations of **1-3** at their E-isomer minima, computed with TDA at the ωB97X-D/6-31G(d) level. The highest state included in the NAMD is S_4. The character of these excitations is further analyzed in Figure S1.1.

	**1**			**2**			**2a**		
State	λ	osc. str.	Type	λ	osc. str.	Type	λ	osc. str.	Type
S_1	423	0.000	nπ*	432	0.000	nπ*	452	0.007	nπ*
S_2	269	0.939	ππ*	293	1.099	ππ*	381	1.159	ππ*
S_3	252	0.016	-	257	0.015	-	325	0.007	-
S_4	230	0.023	-	250	0.002	-	322	0.004	-

State character

1 **2** **2a**

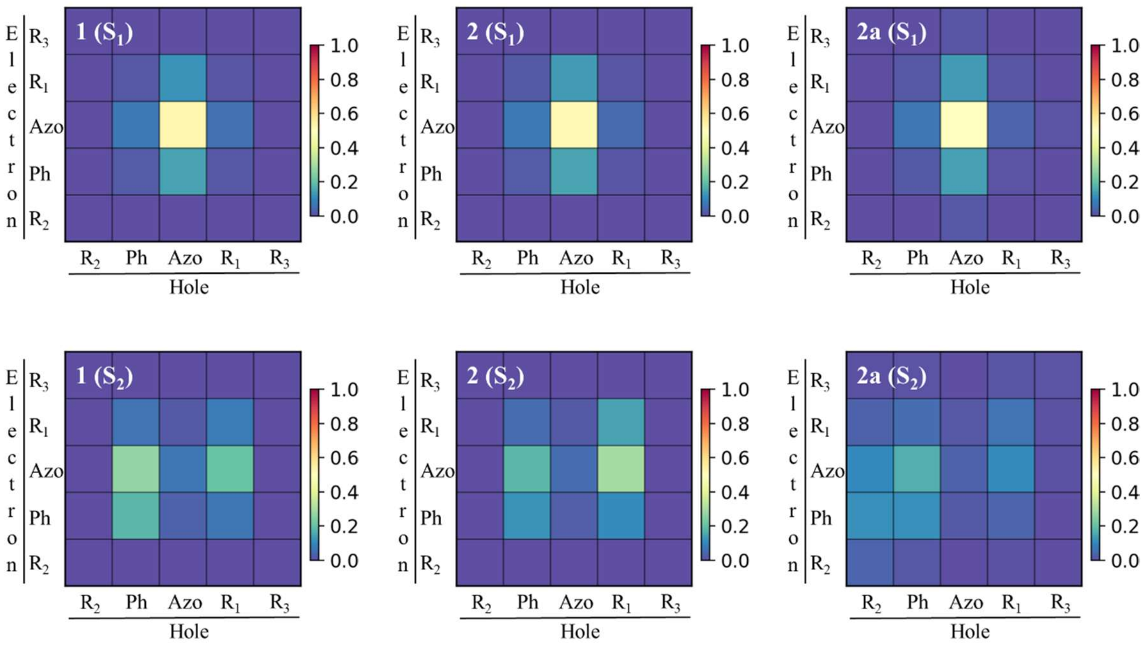

Figure S1.1. Excitation fingerprints associated with the S_1 (top) and S_2 (below) states of **1-2a** obtained with TDA, the ωB97X-D functional, and the 6-31G(d) basis set. R_1 is the Heteroarene, R_2 is the Phenyl substituent and R_3 is the Heteroarene substituent. See below for definition.

The excitation fingerprints (EF) shown above represent the localization of the electron/hole of a given electronic transition (l), using the charge transfer numbers between two molecular fragments (A and B) (Ω_{AB}^{l}). Following our past approach, the molecules were split into five components: the phenyl substituent (R_2), the phenyl (Ph) and azobenzene (Azo) groups, the heteroarene (R_1), and the heteroarene substituent (R_3). As a result, the EF have the form of 5x5 matrices of Ω_{AB}^{l} values (see Figure S1.1), numbered from bottom-left to top-right. The diagonal terms correspond to the energy-transfer components (*i.e.* hole and electron in the same fragment), while the off-diagonal terms are the charge-transfer components of the electronic excitation. The Ω_{AB}^{l} values were obtained from the G09 outputs using *cclib* (for parsing) and the analysis of the transition density matrix provided in TheoDORE version 1.7.1. See ref. [1] for further information on Ω_{AB}^{l}.

S2. Conical Intersection Search and LIIC

Conical Intersections

Table S2.1. Energy (with respect to the E-minima), structure and N=N Wiberg Indices (WI) of CoIn$_A$ and CoIn$_B$, found using CIOPT as the search algorithm, and either ωB97X-D/6-31G(d) within TDA or ADC(2) as the electronic structure methods.

	**CoIn$_A$**						**CoIn$_B$**					
Method	**TDA**			**ADC(2)**			**TDA**			**ADC(2)**		
Compound	**1**	**2**	**2a**	**1**	**2**	**2a**	**1**	**2**	**2a**	**1**	**2**	**2a**
E(kcal/mol)	57.2	54.2	50.2	52.6	46.7	40.5	76.1	78.5	64.2	-	-	-
E(eV)	2.481	2.352	2.177	2.279	2.025	1.756	3.30	3.40	2.784	-	-	-
|CNNC-180|	89.0	87.2	83.1	81.8	77.3	83.1	32.0	1.13	0.9	-	-	-
NNC(Ph)	134.6	121.4	134.6	128.5	117.7	118.5	149.4	157.1	152.4	-	-	-
NNC(Het)	127.0	129.0	124.3	115.9	125.1	121.1	150.3	157.1	152.4	-	-	-
d(N-N)	1.247	1.285	1.268	1.306	1.314	1.341	1.217	1.241	1.243	-	-	-
WI	1.802	1.631	1.634	-	-	-	1.717	1.743	1.740	-	-	-

Wiberg indices have been computed on the CoIn found with TDA using the Natural Bond Order analysis (version 3.1) implemented in Gaussian 09.

The search of minimal energy crossing points with ADC(2) was performed with CIOPT[2] interfaced with Turbomole 7.1.[3] We used the TZVP basis set, the resolution of identity, and the frozen core approximations. ADC(2) has shown good accuracy at treating the excited states of small- and medium-size organic molecules whose ground state does not show static correlation.[4-11] Also, it incorporates by construction the treatment of dynamic correlation, and provide analytical excited-state gradients, which is particularly useful for the search of stationary points. Contrarily to TDA, ADC(2) is too stable close to a CoIn and, as a result, it does not stop at CoIn$_B$, but continues towards CoIn$_A$.

Table S2.2. Energy (with respect to the E-minima) and structure of CoIn$_C$ found using CIOPT as the search algorithm, and either ωB97X-D/6-31G(d) within TDA or ADC(2) as the electronic structure methods. The final structures are however not fully optimized (see comment below), so we also report the S_2-S_1 energy gap.

**CoIn$_C$**			
Method	**TDA**		
Compound	**1**	**2**	**2a**
E(kcal/mol)	116.3	104.8	88.4
E(eV)	5.04	4.54	3.83
S_1-S_0 gap (eV)	0.02	0.25	0.38
|CNNC-180|	0.0	0.5	8.9
NNC(Ph)	100.6	101.9	115.1
NNC(Het)	99.4	103.2	113.3
d(N-N)	1.420	1.367	1.426

Attempts to optimize a non-planar CoIn connecting the S_2 and S_1 states (CoIn$_C$), as proposed in reference 13 of the main text, have been unsuccessful. The CoIn-search method (CIOPT) drives the optimization towards the planar CoIn$_C$ described above. Unfortunately, while CIOPT reduces the energy-gap gradient when approaching the planar CoIn$_C$, in most cases it fails before a reasonably energy gap (~0.01 eV) can be achieved (see Table S2.2). Attempts to improve convergence by tuning internal parameters of the method (σ, α)[2] were unsuccessful. As a result, we cannot provide a fully-optimized planar CoIn$_C$ structure fulfilling the convergence criteria, as we do for CoIn$_A$ and CoIn$_B$, so the structures described in Table S2.2 must be taken with caution. We must highlight that for thiazole-based heteroarenes, it has been reported that the ππ* to nπ* transition is barrierless (*i.e.* without CoIn), occurs at a planar structure, and with a strong vibronic coupling between the two states.[12] Such *avoided-crossing* scenario is in agreement with our results, and explains why the CoIn-search algorithm gets stuck at a point with a large energy gap.

Linear Interpolation of Internal Coordinates (LIIC)

2a

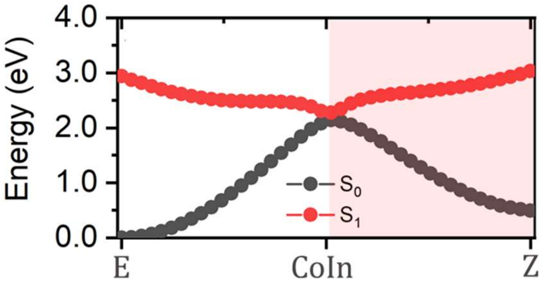

Figure S2.1. Potential Energy Surface of S_0 and S_1 along the coordinate connecting the E- and Z- isomers with $CoIn_A$ computed at the ωB97X-D/6-31G(d) level. The main coordinate is rotation about the CNNC dihedral. The region highlighted in red is (in principle) not explored in our NAMD simulations

S3. Trajectory Analysis

Population

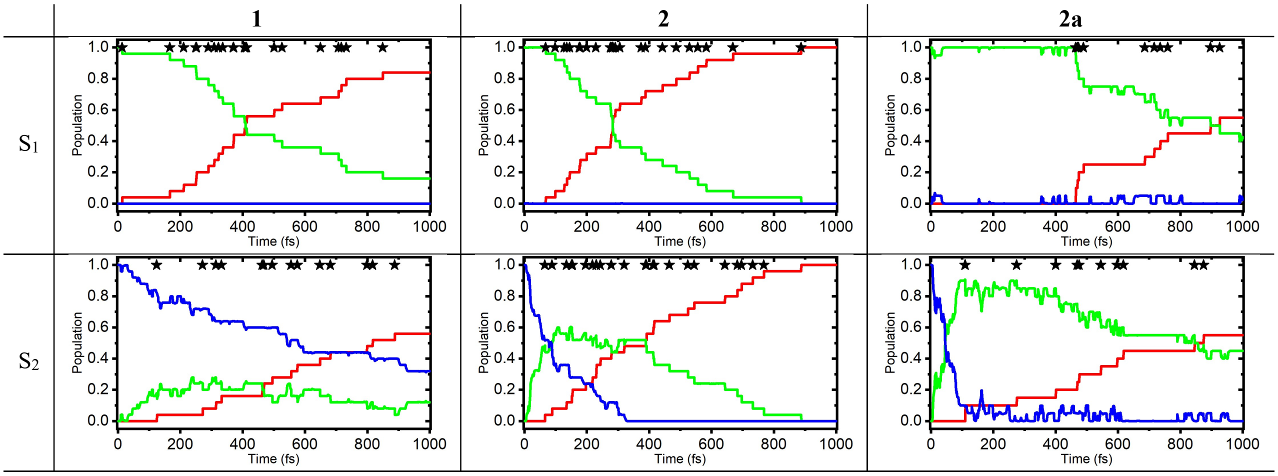

Figure S3.1. Population of the ground state (**red**), the nπ* state (S_1, **green**), and the ππ* state (S_2, **blue**) along the trajectories associated with compounds **1** (left), **2** (middle) and **2a** (right) after excitation to S_1 (top) and S_2 (bottom). It is assumed that S_0 is immediately populated after reaching an S_1/S_0 CoIn. Times at which a trajectory reaches a S_1/S_0 CoIn are marked with stars (★).

Structure of the $n\pi^*_{azo}/S_0$ *Conical Intersections and* S_n *Crossings*

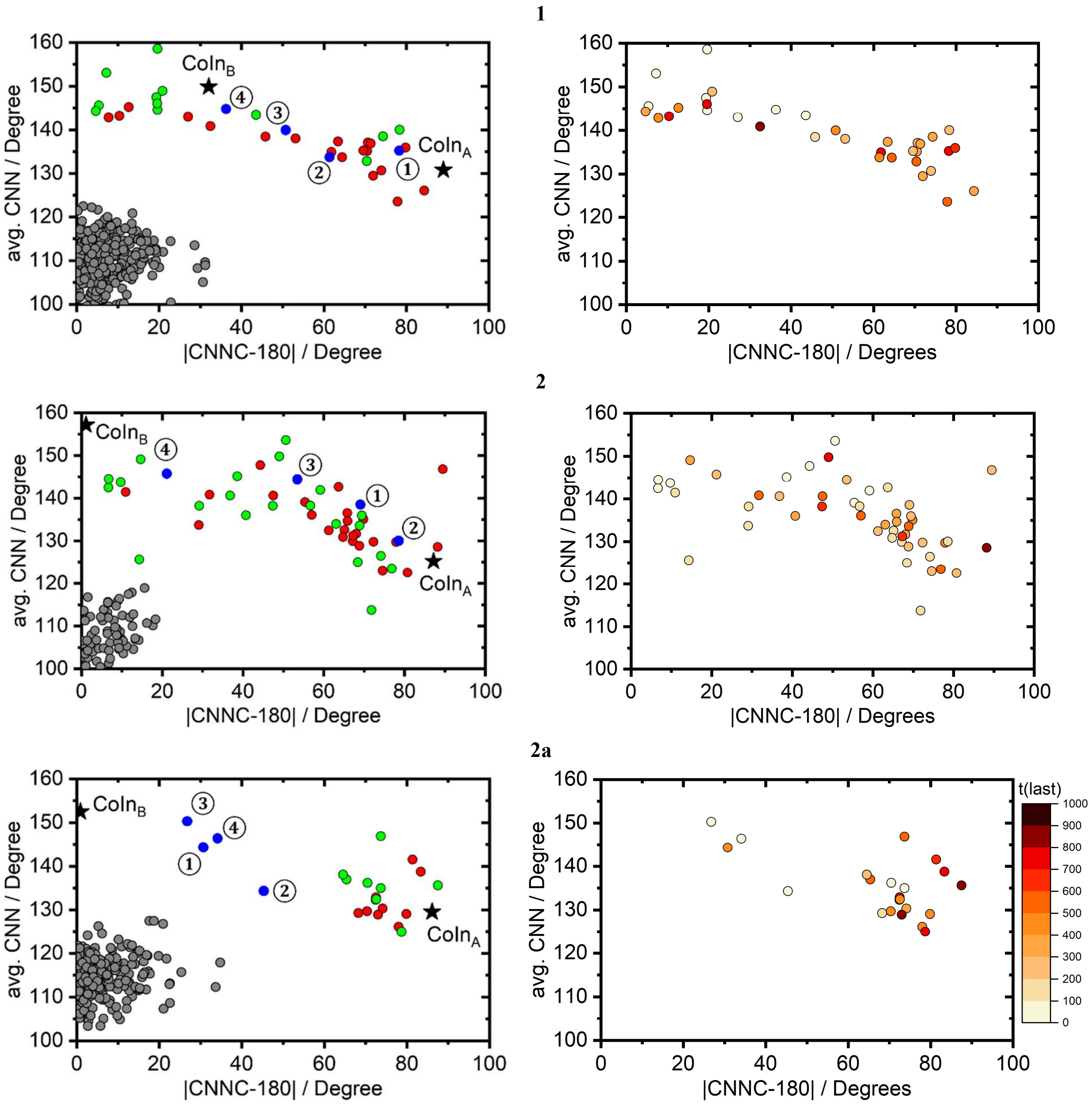

Figure S3.2. Comparison between the (left) space of CNN and CNNC angles featured by the relevant geometries in all trajectories of compounds **1, 2** and **2a** (see Figure 3 for an extended description), and (right) the associated t_{Last} (see color code in the bottom-right figure, in fs). Structures with a more pronounced inversion character (top-left corner) display faster kinetics. The CNNC angle is evaluated as the deviation from planarity, with 0º corresponding to the E-isomer, and 90º corresponding to CNNC either +90 or −90 degrees. Points ①-④ have been recomputed with CC2 and ADC(2) methods in Table S3.2.

Confidence interval

Table S3.1. Range of values associated with a 90% confidence interval and a normal distribution, for the data displayed in Table 1 of the main text. Times are given in fs.

	Initial State	Compound		
		**1**	**2**	**2a**
Ratio	S_1	0.12	0.00	0.18
	S_2	0.16	0.06	0.18
t_{CoIn}	S_1	76	65	89
	S_2	98	72	126
t_{S1}	S_1	76	65	83
	S_2	101	64	118
t_{S2}	S_1	-	-	10
	S_2	89	33	29
t_{Last}	S_1	76	65	103
	S_2	102	64	148

Convergence

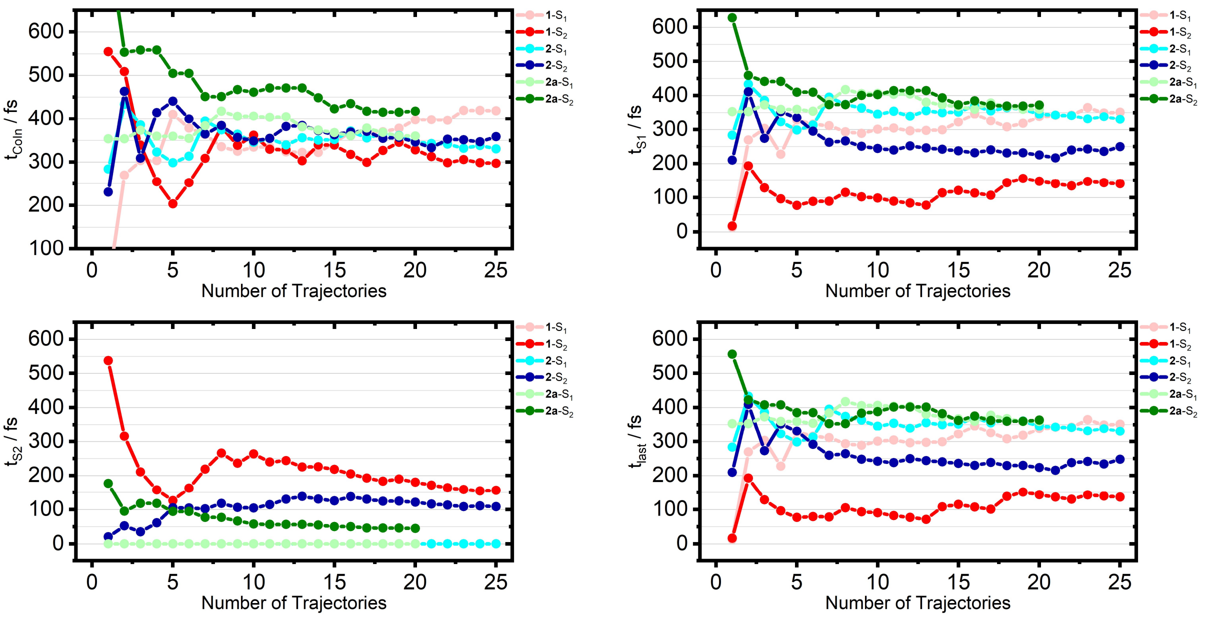

Figure S3.3. Convergence of characteristic times with the number of trajectories run. Only trajectories having reached a CoIn count towards these averages, hence not all trajectories update the values.

Crossing Seam

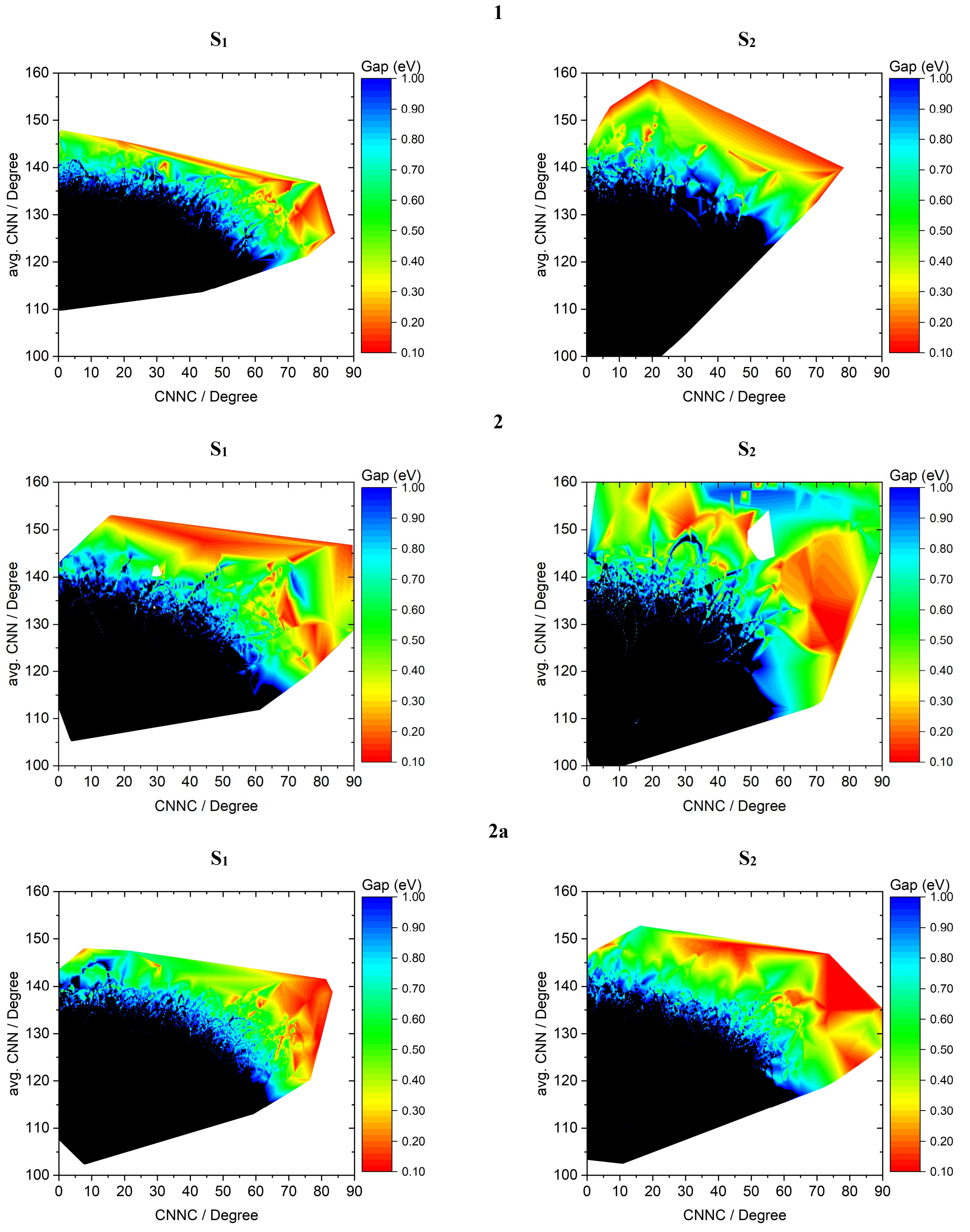

Figure S3.4. Energy gap between S_1 and S_0 along the space of CNN and CNNC angles explored by the NAMD trajectories of compounds **1, 2** and **2a** upon excitation to the S_1 and S_2 states. The coloured (white) region implies that it has (not) been explored at some point by the trajectories. Notice that excitation to S_2 leads to the exploration of a broader region of CNN and CNNC angles. Regions that are more often explored show a finer resolution of points. The region close to the crossing seam is scarcely since the trajectories reach the energetic criterion for termination. As such, the resolution is poor and artefacts appear. Figure **S3.5** shows a different version of this figure with a different colour code.

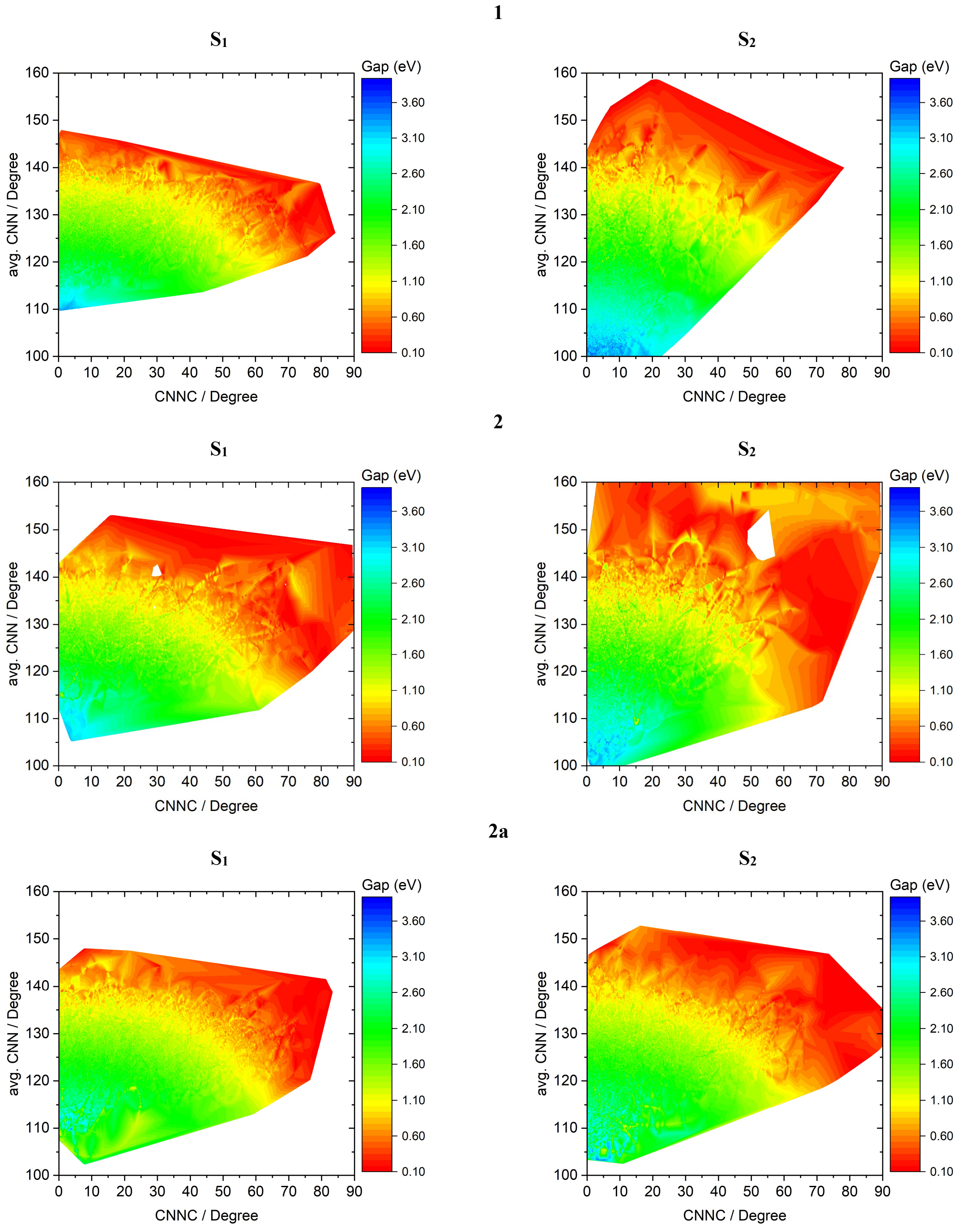

Figure S3.5. Energy gap between S_1 and S_0 along the space of CNN and CNNC angles explored by the NAMD trajectories of compounds **1, 2** and **2a** upon excitation to the S_1 and S_2 states. The coloured (white) region implies that it has (not) been explored at some point by the trajectories. Notice that excitation to S_2 leads to the exploration of a broader region of CNN and CNNC angles. Regions that are more often explored show a finer resolution of points. The region close to the crossing seam is scarcely since the trajectories reach the energetic criterion for termination. As such, the resolution is poor and artefacts appear. Figure **S3.4** shows a different version of this figure with a different colour code.

Table S3.2. Comparison of S_1-S_0 energy gaps (in eV) at selected geometries close to the crossing seam, labelled ①-④ to facilitate identification in Figure S3.2. Geometries with a TDA energy below 0.1 eV are the final step of a NAMD trajectory, at which the energy gap termination criterion is fulfilled. Geometries with a larger TDA energy are from the precedent step. Each geometry corresponds to a different NAMD trajectory. Methods are TDA at the ωB97X-D/6-31G(d) level, and ADC(2) and CC2 methods with the TZVP basis set.

	CNN	|CNNC-180|	Point	TDA	ADC(2)	CC2
**1**	135.2	78.2	①	0.09	0.10	0.17
**1**	133.8	61.4	②	0.07	0.26	0.29
**1**	139.9	50.8	③	0.22	0.43	0.45
**1**	144.7	36.2	④	0.10	0.24	0.28
**2**	138.5	69.0	①	0.47	0.42	0.52
**2**	140.6	47.5	②	0.23	0.40	0.45
**2**	129.7	77.9	③	0.03	0.01	0.14
**2**	149.1	14.6	④	0.39	0.33	0.44
**2a**	144.3	30.7	①	0.05	0.16	0.18
**2a**	134.3	45.3	②	0.40	0.42	0.51
**2a**	150.2	26.8	③	0.27	0.27	0.32
**2a**	146.4	34.1	④	0.15	0.00	0.06

Energy Conservation

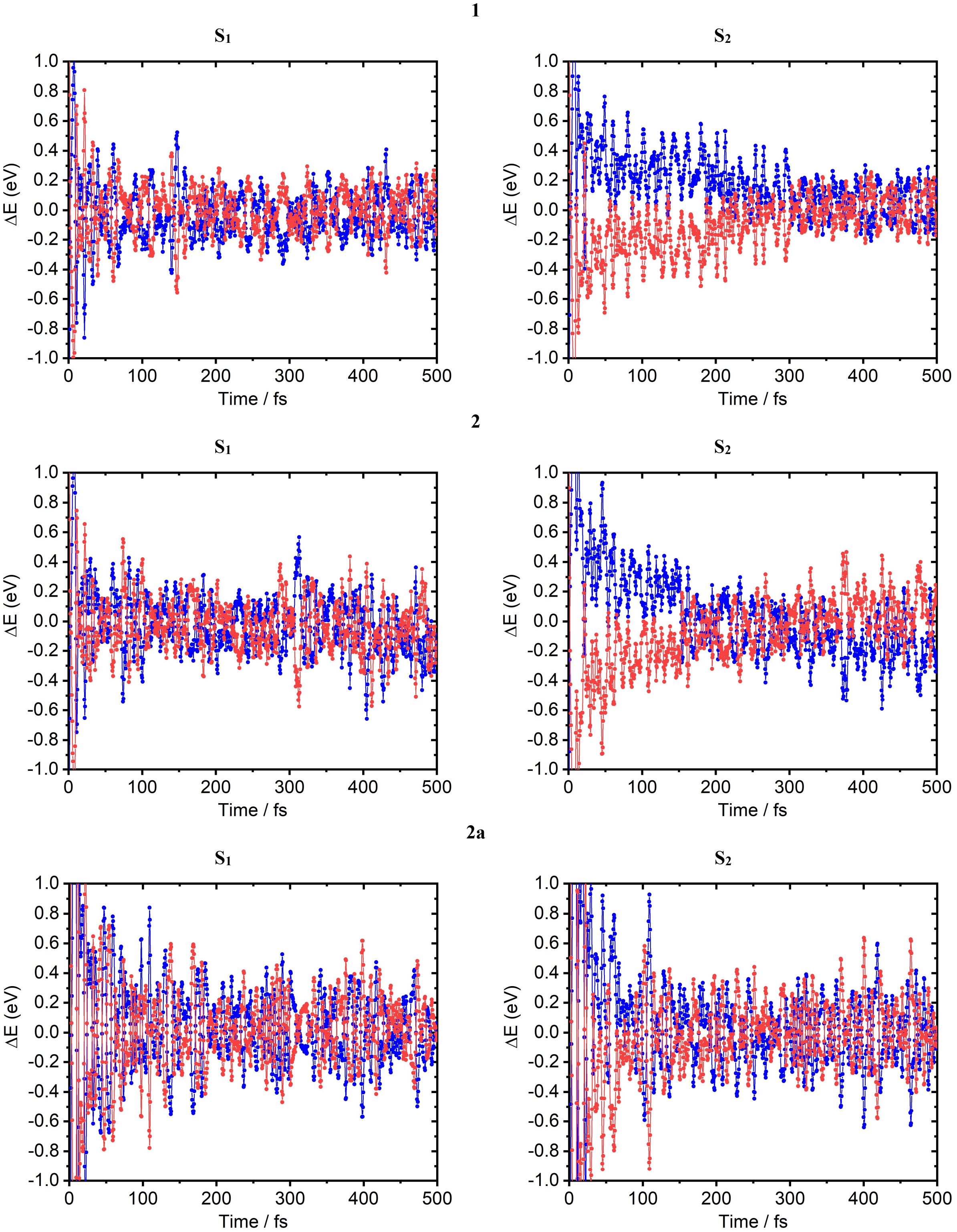

Figure S3.6. Evolution of the potential (blue) and kinetic (red) energy with respect to the average for each ensemble of trajectories during the first 500 fs. The total energy (potential + kinetic) is conserved in each trajectory below the default value of 0.5 eV.

References

1. Mai, S.; Plasser, F.; Dorn, J.; Fumanal, M.; Daniel, C.; González, L. Quantitative wave function analysis for excited states of transition metal complexes. *Coord. Chem. Rev.* **2018,** 361, 74-97.

2. Levine, B. G.; Coe, J. D.; Martínez, T. J. Optimizing Conical Intersections without Derivative Coupling Vectors: Application to Multistate Multireference Second-Order Perturbation Theory (MS-CASPT2). *J. Phys. Chem. B* **2008,** 112, 405-413.

3. Furche, F.; Ahlrichs, R.; Hättig, C.; Klopper, W.; Sierka, M.; Weigend, F. Turbomole. *Wiley Interdisciplinary Reviews: Computational Molecular Science* **2014,** 4, 91-100.

4. Hättig, C. In *Adv. Quantum Chem.*; Jensen, H. J. Å., Ed.; Academic Press: 2005; Vol. 50, pp 37-60.

5. Tuna, D.; Lefrancois, D.; Wolański, Ł.; Gozem, S.; Schapiro, I.; Andruniów, T.; Dreuw, A.; Olivucci, M. Assessment of Approximate Coupled-Cluster and Algebraic-Diagrammatic-Construction Methods for Ground- and Excited-State Reaction Paths and the Conical-Intersection Seam of a Retinal-Chromophore Model. *J. Chem. Theory Comput.* **2015,** 11, 5758-5781.

6. Winter, N. O. C.; Graf, N. K.; Leutwyler, S.; Hättig, C. Benchmarks for 0–0 transitions of aromatic organic molecules: DFT/B3LYP, ADC(2), CC2, SOS-CC2 and SCS-CC2 compared to high-resolution gas-phase data. *Phys. Chem. Chem. Phys.* **2013,** 15, 6623-6630.

7. Jacquemin, D.; Duchemin, I.; Blase, X. 0–0 Energies Using Hybrid Schemes: Benchmarks of TD-DFT, CIS(D), ADC(2), CC2, and BSE/GW formalisms for 80 Real-Life Compounds. *J. Chem. Theory Comput.* **2015,** 11, 5340-5359.

8. Prlj, A.; Sandoval-Salinas, M. E.; Casanova, D.; Jacquemin, D.; Corminboeuf, C. Low-Lying ππ* States of Heteroaromatic Molecules: A Challenge for Excited State Methods. *J. Chem. Theory Comput.* **2016,** 12, 2652-2660.

9. Azarias, C.; Habert, C.; Budzák, Š.; Blase, X.; Duchemin, I.; Jacquemin, D. Calculations of n→π* Transition Energies: Comparisons Between TD-DFT, ADC, CC, CASPT2, and BSE/GW Descriptions. *J. Phys. Chem. A* **2017,** 121, 6122-6134.

10. Suellen, C.; Garcia Freitas, R.; Loos, P.-F.; Jacquemin, D. Cross Comparisons Between Experiment, TD-DFT, CC, and ADC for Transition Energies. *J. Chem. Theory Comput.* **2019**.

11. Loos, P.-F.; Jacquemin, D. Evaluating 0–0 Energies with Theoretical Tools: A Short Review. *ChemPhotoChem* **2019,** 3, 684-696.

12. Garcia-Amorós, J.; Maerz, B.; Reig, M.; Cuadrado, A.; Blancafort, L.; Samoylova, E.; Velasco, D. Picosecond Switchable Azo Dyes. *Chem. Eur, J.* **2019,** 25, 7726-7732.
